# “Not flying solo”: phylogenetic identification and life-cycle insights of larval cestodes in the European flying squid *Todarodes sagittatus* (Cephalopoda: Ommastrephidae)

**DOI:** 10.1016/j.ijppaw.2026.101272

**Published:** 2026-08-10

**Authors:** Nourreddine Benyahia, Chahinez Bouguerche, Nadia Kechemir-Issad

**Affiliations:** a Laboratoire Biodiversité et Environnement: Interactions, Génomes. Equipe Parasites: Biodiversité- Bioécologie-Interactions Hôtes-Parasites, Faculté des Sciences Biologiques, Université des Sciences et de la Technologie Houari Boumedienne, USTHB, BP 32, El-Alia, Bab-Ezzouar, Algiers, 16111, Algeria; b FSAVB. Département de Biologie. Université Saad Dahlab, Route de Soumaa. BP 270, Blida, Algeria; c Department of Zoology, Swedish Museum of Natural History, Box 50007, Stockholm, SE-104 05, Sweden

**Keywords:** *Todarodes sagittatus*, *Crossobothrium*, Life cycle, Plerocercoid, Mediterranean, Molecular barcoding

## Abstract

The European flying squid *Todarodes sagittatus* Lamarck is a widely distributed ommastrephid cephalopod in the Northeast Atlantic and Mediterranean Sea, yet its parasite fauna remains poorly documented in the Mediterranean Sea. In this study, two cestode larvae species infecting *T. sagittatus* from the Algerian coast (Western Mediterranean) were investigated using an integrative approach combining morphological observations and molecular analyses. A total of 63 squids were examined for parasitic infection. Cestodes in plerocercoid stage were detected in 31 individuals (prevalence of 46%), primarily located within the gastrointestinal tract, including the stomach, intestine, and caecum. Morphological features of the larvae were consistent with members of the order Phyllobothriidea but did not allow identification to the species level. Molecular analysis of the D1–D3 region of the 28S rDNA revealed two distinct larval lineages belonging to the genus *Crossobothrium* Linton, 1889. One lineage showed 100% sequence identity with the adult cestode *Crossobothrium dohrnii* (Oerley, 1885), a parasite of hexanchid sharks, confirming the identity of these larvae as plerocercoids of *C. dohrnii*. The second lineage clustered within the *Crossobothrium* clade but could not be assigned to a known species and is therefore referred to as *Crossobothrium* sp. The occurrence of these larvae in *T. sagittatus*, together with ecological data on squid diet and predator-prey relationships, suggests that this cephalopod acts as an intermediate or paratenic host in the life cycle of hexanchid cestodes. Infection likely occurs through predation on crustaceans acting as first intermediate hosts, while transmission to definitive hosts occurs when infected squids are consumed by sharks. These findings provide the first molecular identification of cestode larvae from *T. sagittatus* in the Mediterranean and highlight the important role of ommastrephid squids in the trophic transmission of elasmobranch parasites in pelagic ecosystems.

## Highlights

• First molecular identification of *Crossobothrium* larvae in *Todarodes sagittatus.*
• *Crossobothrium dohrnii* plerocercoids were confirmed by 28S rDNA.
• A second *Crossobothrium* lineage was detected in western Mediterranean *T. sagittatus.*
• *Todarodes sagittatus* likely acts as an intermediate or paratenic host.
• Ommastrephid squids may link cestodes to hexanchid sharks.

## Graphical abstract

**Figure:**
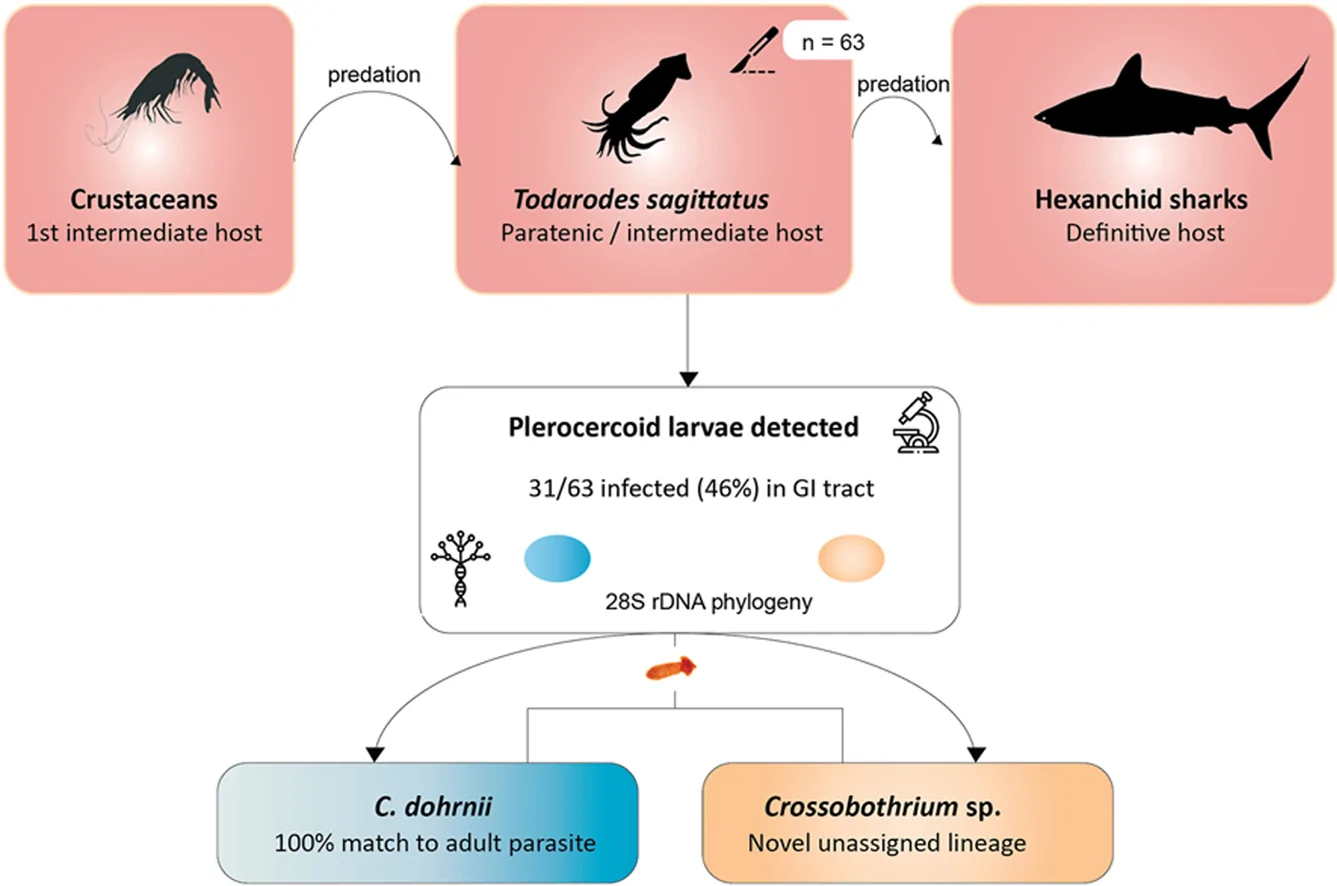

## 1 Introduction

The European flying squid *Todarodes sagittatus* Lamarck (Ommastrephidae) is widely distributed in the Northeast Atlantic and throughout the Mediterranean Sea (Jereb and Roper, 2010). Most studies on this species have been conducted within its Atlantic range (Rosenberg et al., 1981; Breiby and Jobling, 1985; Piatkowski et al., 1998; Lordan et al., 2001), whereas it has received considerably less attention in the Mediterranean (Quetglas et al., 1998, 1999; Rosas-Luis et al., 2014). In Algeria, *T. sagittatus* is regularly captured as bycatch during trawling operations but has never been the subject of dedicated investigations, either regarding its biology and ecology or within the context of fisheries management. Its parasite fauna also remains unexplored, despite the importance of parasitological studies for understanding trophic interactions and monitoring seafood safety.

Where investigated, *T. sagittatus* has been shown to harbour a diverse assemblage of parasites. These include third-stage larvae of nematodes of the genus *Anisakis* (Abollo et al., 1998; Farjallah et al., 2008; Angelucci et al., 2011; Cipriani et al., 2021), which may cause anisakiasis in humans; coccidians of the genus *Aggregata* Frenzel, 1885 (Gestal et al., 2000); trematodes (Overstreet and Hochberg, 1975; Vidal and Haimovici, 1999), and larval cestodes (Hochberg, 1990). Plerocercoid larvae have been reported in several squid species of the genus *Todarodes* Steenstrup. In the Japanese flying squid *T. pacificus* (Steenstrup), larvae identified as *Scolex pleuronectis* Müller, 1788 and *Orygmatobothrium* sp. have been recorded (Kim et al., 1999). In the Angolan flying squid *T. angolensis* Adam from the southeast Atlantic, records include *Phyllobothrium* sp. and *Tentacularia coryphaenae* Bosc, 1802 (Gaevskaya and Nigmatullin (1975), 1978), whereas *S*. *pleuronectis* has been reported in *T. sagittatus* from Atlantic waters (Dollfus, 1936; Nigmatullin et al., 2008).

Cephalopods occupy a key position in marine food webs (Rohde, 2002). As important prey for numerous teleosts, chondrichthyans, cetaceans and seabirds (Coll et al., 2013), they contribute significantly to the transmission of trophically transmitted parasites, including cestodes, within marine ecosystems. Consequently, cephalopods often act as intermediate or paratenic hosts in the life cycles of several helminths (Roumbedakis et al., 2018). Global syntheses of parasite records have further highlighted the remarkable but still underestimated diversity of parasites associated with cephalopods and emphasized the need for targeted parasitological studies on poorly investigated hosts and regions (Tedesco et al., 2020). Most cestodes reported from cephalopods correspond to larval stages belonging to the polyphyletic order “Tetraphyllidea” and to the order Trypanorhyncha (Hochberg, 1990; Kuchta et al., 2008). However, the identification of these larvae is often problematic because cestode taxonomy is largely based on morphological features of adult worms (Euzet, 1994), which are absent or poorly developed in larval stages. This difficulty is particularly pronounced in members of the former “Tetraphyllidea”. By contrast, trypanorhynch larvae can generally be identified more reliably owing to the presence of a characteristic rhyncheal apparatus that allows identification at least to the genus level (Palm and Schröder, 2001). Hence, molecular approaches have increasingly been employed to assist in the identification of larval cestodes. Genetic markers such as 28S rDNA, 18S rDNA, ITS regions and mitochondrial genes (e.g. *cox*1) have proven particularly useful for linking larval stages to their adult counterparts and clarifying phylogenetic relationships (Caira et al., 2014; Kuchta et al., 2013; Scholz and Kuchta, 2022). Molecular studies conducted on metacestodes from marine hosts including cephalopods have demonstrated the effectiveness of combining morphological and molecular data for resolving the identity of larval cestodes and improving our understanding of their life cycles (Jensen and Bullard, 2010; Guardone et al., 2020).

Despite these advances, information on cestode larvae infecting *T. sagittatus* remains limited. Previous parasitological investigations have reported plerocercoids identified as *Phyllobothrium* sp., the trypanorhynch *Nybelinia* sp., and *Scolex pleuronectis* (Squires, 1957; Threlfall, 1970; Stunkard, 1977; Gaevskaya and Nigmatullin, 1978; Bower and Margolis, 1991). In the Mediterranean Sea, however, records remain scarce and largely historical (Monticelli, 1888; Dollfus, 1958), highlighting the need for updated studies integrating morphological and molecular approaches. Thus, the present study aims to identify cestode larvae recovered from the digestive tract of *T. sagittatus* using an integrative approach combining morphological observations and molecular analyses, and to elucidate the role of this squid in the life cycle of marine cestodes.

## 2 Materials and methods

### 2.1 Sampling

A total of 63 specimens of *T. sagittatus* were obtained from local fish markets. These specimens originated from fishing grounds off the Algerian coast near Algiers, Western Mediterranean (36°47′00″ N, 3°09′00″ E) and were collected between October 2002 and September 2005. All specimens were examined fresh. The size and sex of each individual were recorded prior to dissection. Each squid was dissected longitudinally along the ventral surface and carefully examined under a stereomicroscope. All cestode larvae encountered were collected and counted. Infection parameters, including prevalence (P), intensity (I), and mean intensity (MI), were calculated according to the definitions provided by Bush et al. (1997). Geographical designations follow the FAO Major Fishing Areas classification (https://www.fao.org/fishery/en/area/search), to ensure standardized naming of marine regions.

### 2.2 Fixation, staining, and morphological observation of larvae

Most of the recovered cestode larvae were fixed in aqueous Bouin's solution^1^for morphological examination, while a subset of 11 larvae was preserved in 95% ethanol for molecular analyses. Specimens intended for morphological study were stained with boracic carmine or acetic carmine. After staining, the larvae were dehydrated through a graded ethanol series (70%, 95%, and 100%), cleared in eugenol, and permanently mounted in Canada balsam between glass slides and coverslips. The preparations were subsequently dried in an oven at 60 °C. Morphological observations were performed using a Zeiss Axioplan light microscope at various magnifications. Digital images were captured using the Zeiss AxioVision imaging system, which was also used to obtain morphometric measurements of the larvae. Differences in infection prevalence between male and female squids were assessed using a chi-square test with a significance threshold of *p* = 0.05. Statistical analyses were performed using Microsoft Excel 2016.

### 2.3 Molecular analysis

Total genomic DNA was individually extracted from 11 plerocercoid larvae preserved in 95% ethanol using the QIAamp® DNA Micro Kit (QIAGEN, Germany); a fraction of each specimen was used for extraction while the remainder was kept as a voucher, so that these larvae represent paragenophores *sensu* Pleijel et al. (2008). Molecular analysis of the 11 larvae revealed only two distinct molecular signatures, corresponding to two cestode taxa/genotypes. The D1–D3 domain of the nuclear large subunit ribosomal RNA gene (28S rDNA), approximately 1300 bp in length, was amplified by polymerase chain reaction (PCR) using the primers LSU5 and 1200R. Each PCR reaction consisted of 10 μL of TP5X buffer, 0.5 μL of Green GoTaq Master Mix (Promega, Madison, WI, USA), 3.75 μL of each primer (5 μM), 1 μL of dNTPs, 23.5 μL of sterile distilled water, and 3 μL of genomic DNA, yielding a final reaction volume of 46 μL. PCR amplifications were performed using a PTC-100 thermocycler (Bio-Rad) with the following cycling conditions: initial denaturation at 96 °C for 2 min 30 s; 40 cycles of denaturation at 96 °C for 30 s, annealing at 62 °C for 20 s, and extension at 72 °C for 40 s; followed by a final extension at 72 °C for 90 s. Amplified products were separated on a 1.5% agarose gel. DNA concentrations were estimated using a 100 bp DNA ladder (Promega). PCR products were purified using the Wizard SV Gel and PCR Clean-Up System (Promega) according to the manufacturer's protocol. Sequencing reactions were performed using the GenomeLab DTCS-Quick Start Kit (Beckman Coulter). In addition to the amplification primers, internal primers ECD2 and 300F were used to sequence the central region of the D1–D3 domain, including the variable D2 region (Table 1). Sequencing was conducted using the dye-terminator method on an automated CEQ 8000 sequencer (Beckman Coulter). Contiguous sequences were assembled and edited using Sequencher 4.5™ (Gene Codes Corp., Ann Arbor, MI, USA). Non-overlapping regions at both ends of the sequences were trimmed prior to analysis. The resulting sequences were compared with those available in the NCBI nucleotide database using the BLAST (McGinnis and Madden, 2004). The newly generated sequences were deposited in GenBank under accession numbers KU359304–KU359314 (see Table 2).

**Table 1:** Primers used for PCR amplification and sequencing of the 28S rDNA, with primer orientation and reference (Littlewood et al., 2000).

Primer	Sequence (5′–3′)	Direction
LSU5	TAGGTCGACCCGCTGAAYTTAAGC	Forward
1200R	GCATAGTTCACCATCTTTCGG	Reverse
300F	CAAGTACCGTGAGGGAAAGTT	Forward
ECD2	CTTGGTCCGTGTTTCAAGACGGG	Reverse

**Table 2:** Cestode taxa retrieved from GenBank and included in the phylogenetic analysis, with host species, sampling locality and GenBank accession numbers. Newly generated sequences are in bold blue.

Taxon	Host	Locality	GenBank Accession	References
*Clistobothrium montaukensis*	*Isurus oxyrinchus*	Toshima Island, Japan, NWP	LC195130-33	Unpublished

*Clistobothrium montaukensis*	*Isurus oxyrinchus*	USA	AF286957	Olson et al. (2001)

*Clistobothrium montaukensis* (larval cestode)	*Regalecus glesne*	Coast of Santa Catalina Island, California, ECP	KM272991	Kuris et al. (2015)

*Clistobothrium montaukensis*	*Isurus oxyrhynchus*	Montauk, New York, WCA	EF095259	Waeschenbach et al. (2007)



*Clistobothrium montaukensis*	*Isurus oxyrinchus*	Fugang, Taiwan, WCP	MT732122-23	Caira et al., 2020a

		Montauk, New York, WCA	MT732124, 25	Caira et al., 2020b

*Clistobothrium montaukensis*	*Isurus oxyrinchus*	Ogasawara Islands, Japan, NWP	LC195134	Unpublished



*Clistobothrium* sp.	*Illex argentinus*	Argentina	OR725127-32	Gutiérrez et al. (2023)

*Clistobothrium* sp.	*Regalecus glesne*	Coast of Santa Catalina Island, California, ECP	KM272992	Kuris et al. (2015)

*Clistobothrium* sp. n. 1	*Alopias vulpinus*	New Bedford, Massachusetts, NWA	MT732134	Caira et al., 2020a

*Pelichnibothrium speciosum*	*Alepisaurus ferox*	Toshima Island, Japan, NWP	LC195126	Unpublished

*Pelichnibothrium speciosum*	*Prionace glauca*	Tosashimizu, Japan, NWP	LC195128	Unpublished

*Clistobothrium* sp. n. 2	*Isurus paucus*	Nanfang-ao Taiwan, NWP	MT732130	Caira et al., 2020b



*Clistobothrium* sp. n. 3	*Onchorhynchus nerka*	Alaska, NEP	MT732126-29	Caira et al., 2020a

*Clistobothrium* sp. 3	*Oncorhynchus keta*	Sanduga River, Russia	OR148444	Unpublished



*Clistobothrium delphini*	*Globicephala melas*	Spain, WM	OR886384	Specht et al. (2024)

*Clistobothrium grimaldii*	*Arctocephalus pusillus*	Japan (Zoo)	LC718556	Unpublished

*Clistobothrium* sp.	*Arctocephalus pusillus*	Germany (Zoo), caught in South Africa	KU724058	Klotz et al. (2018)

*Clistobothrium grimaldii*	*Globicephala melas*	Spain, WM	OR886385	Specht et al. (2024)

*Phyllobothriid* sp. 1	*Stenella coeruleoalba*	Spain, WM	DQ839568	Aznar et al. (2007)

*Clistobothrium tumidum*	*Carcharodon carcharias*	Cape May, New Jersey, NWA	MT732142, 43	Caira et al., 2020b

*Clistobothrium gabywalterorum*	*Pseudocarcharias kamoharai*	Santa Rosa de Salinas, Ecuador, SEP	MN706183	[Bibr bib14][Bibr bib15]

Phyllobothriidae sp.	*Gymnscopelus nicholsi*	Agujero Azul, Argentina, SWA	PP085582	Timi et al. (2024)

*Clistobothrium* sp.	*Hexanchus griseus*	Mediterranean Sea	PP153400	Lopez-Verdejo et al. (2024)

*Thysanocephalum thysanocephalum*	*Galeocerdo cuvier*	South Carolina, NWA	KF685902	Caira et al. (2014)

*Thysanocephalum* sp.	*Galeocerdo* cuvier	USA	AF286963	Olson et al. (2001)

*Guidus* sp.	*Bathyraja multispinis*	Falkland Islands, SWA	MH688710	Beer et al. (2019)

Tetraphyllidea fam. gen. sp.	*Alepocephalus rostratus*	NW Mediterranean	KP249697, KP249698	Pérez-i-García et al. (2015)

*Calyptrobothrium* sp. 1	*Torpedo nobiliana*	Rhode Island, NWA	KF685754	Caira et al. (2014)

*Yamaguticestus longicollis*^a^	*Scyliorhinus canicula*	UK, NEA	AF286958	Olson et al. (2001)

*Yamaguticestus* cf. *squali*	*Squalus acanthias*	Balchik, Bulgaria, Black Sea	MW419976	Caira et al., 2021a

*Yamaguticestus squali*	*Squalus acanthias*	Rhode Island, NWA	KF685897	Caira et al. (2014)

*Yamaguticestus* sp.	*Scyliorhinus canicula*	North Sea, NEA	MZ353646	Caira et al. (2021b)

*Yamaguticestus squali*	*Squalus acanthias*	Rhode Island, NWA	KC543441	Ruhnke and Workman (2013)

*Yamaguticestus fairweatherae*^b^	*Holohalaelurus regani*	South Africa, WIO	MZ353647	Caira et al. (2021b)

*Yamaguticestus africanae*^c^	*Scyliorhinus capensis*	South Africa, WIO	MZ353645	Caira et al. (2021b)

*Yamaguticestus kuchtai*	*Apristurus laurussonii*	UK, NEA	PV972202	Higueruelo et al. (2026)

*Yamaguticestus kuchtai*	*Apristurus aphyodes*	UK, NEA	PV972203	Higueruelo et al. (2026)

*Calyptrobothrium* sp.	*Apristurus atlanticus*	UK, NEA	AF382087	Brickle et al. (2001)

Phyllobothriidea ‘gen n. sp. 2’	*Bathyraja albomaculata*	Falkland Islands, SWA	MH688715	Beer et al. (2019)

Rockacestus sp. n. 5	*Amblyraja doellojuradoi*	Falkland Islands, SWA	MW419961	Caira et al., 2021bc

Phyllobothriidea ‘gen n. sp. 2’	*Bathyraja multispinis*	Falkland Islands, SWA	MH688712	Beer et al. (2019)

Phyllobothriidea ‘gen n. sp. 2’	*Bathyraja macloviana*	Falkland Islands, SWA	MH688714	Beer et al. (2019)

Phyllobothriidea ‘gen n. sp. 2’	*Bathyraja brachyurops*	Falkland Islands, SWA	MH688717	Beer et al. (2019)

Phyllobothriidea ‘gen n. sp. 2’	*Amblyraja doellojuradoi*	Falkland Islands, SWA	MH688716	Beer et al. (2019)

*Rockacestus conchai*	*Bathyraja albomaculata*	Falkland Islands, SWA	MW419959	Caira et al., 2021bc

Phyllobothriidea ‘gen n. sp. 1’	*Raja montagui*	North Sea, NEA	MH688711	Beer et al. (2019)

Phyllobothriidea ‘gen n. sp. 3’	*Bathyraja cousseauae*	Falkland Islands, Atlantic Ocean	MH688720	Beer et al. (2019)

Phyllobothriidea ‘gen n. sp. 3’	*Dipturus chilensis*	Falkland Islands, Atlantic Ocean	MH688718	Beer et al. (2019)

Phyllobothriidea ‘gen n. sp. 3’	*Bathyraja magellanica*	Falkland Islands, Atlantic Ocean	MH688719	Beer et al. (2019)

*Rockacestus piriei*	*Gasterosteus aculeatus*	Kandalakshsky Bay, Russia White Sea	PV382413	Biserova et al. (2025)

*Rockacestus piriei*	*Leucoraja naevus*	North Sea, NEA	MH688721	Beer et al. (2019)

Phyllobothriidea ‘gen n. sp. 3’	*Psammobatis* sp. 3	Falkland Islands, SWA	MH688722, MH688723	Beer et al. (2019)

*Rockacestus piriei*	*Gasterosteus aculeatus*	Kandalakshsky Bay, Russia White Sea	PV382412	Biserova et al. (2025)

*Phyllobothrium* cf. *lactuca*	*Mustelus mento*	Puerto Montt, Chile, SEP	KF685770	Caira et al. (2014)

Phyllobothriidea ‘gen. n. 10 sp. n. 1’	*Sphyrna lewini*	Florida, NWA	KF685889	Caira et al. (2014)

*Myzophyllobothrium myliobatidis*^d^	*Aetomylaeus maculatus*	Sarawak, Malaysia, WCP	MZ189007	Jensen et al. (2021)

*Myzophyllobothrium myliobatidis*	*Aetomylaeus maculatus*	Sarawak, Malaysia, WCP	MZ189008	Jensen et al. (2021)

*Myzophyllobothrium* cf. *gambangi*^e^	*Stegostoma fasciatum*	Sarawak, Malaysia, WCP	KF685774	Caira et al. (2014)

*Myzophyllobothrium gambangi*^f^	*Aetomylaeus nichofii*	South Kalimantan, Indonesia, WCP	MZ189009	Jensen et al. (2021)

*Myzophyllobothrium* sp. 'cf. *nagasawai*'	*Aetobatus narutobiei*	Cat Ba Island, VietNam, WCP	MZ189005 MZ189006	Jensen et al. (2021)

*Myzophyllobothrium* sp. g	*Aetobatus ocellatus*	Darwin, Northern Territory, Australia, WCP	KF685887	Caira et al. (2014)

*Myzophyllobothrium narinari*^h^	*Aetobatus ocellatus*	West Kalimantan, Indonesia, WCP	MZ189002	Jensen et al. (2021)

*Myzophyllobothrium narinari*^h^	*Aetobatus ocellatus*	Gulf of Mannar, Sri Lanka, EIO	MZ189003-04	Jensen et al. (2021)



*Phoreiobothrium lewinense*	*Sphyrna lewini*	Florida, NWA	KF685896	Caira et al. (2014)

*Phoreiobothrium* sp.	*Opsanus beta*	Gulf of Mexico, WCA	GQ470062	Jensen and Bullard (2010)

*Triloculatum andersonorum*	*Negaprion acutidens*	Weipa, Queensland, Australia, WCP	KF685895	Caira et al., 2014

*Triloculatum* sp.	*Ariopsis felis*	Gulf of Mexico, WCA	GQ470100	Jensen and Bullard (2010)

*Trilocularia acanthiaevulgaris*^i^	*Squalus acanthias*	Rhode Island, NWA	KF685776	Caira et al. (2014)

*Trilocularia acanthiaevulgari*	*Squalus acanthias*^j^	Russia	PQ867141-43	Unpublished



*Crossobothrium laciniatum*	*Hexanchus griseus*	Puerto Montt, Chile, SEP	KF685883	Caira et al. (2014)
Tetraphyllidea sp. 2	*Todarodes sagittatus*	Algiers, Algeria, WM	KU359311- 14	Unpublished
*Crossobothrium* cf. *dohrnii*	*Heptranchias perlo*	Gulf of Mexico, WCA	KF685759	Caira et al. (2014)
*Crossobothrium* sp.	*Carcharias taurus*	Moreton Bay, Queensland, Australia, WCP	MG008945	Cutmore et al. (2017)

**Tetraphyllidea sp. 1**	***Todarodes sagittatus***	**Algiers, Algeria, WM**	**KU359304-10**	**Present study**
*Crossobothrium dohrnii*	*Hexanchus griseus*	Mediterranean Sea	PP153402	Lopez-Verdejo et al. (2024)

Abbreviations: Abbreviations: NWA, Northwest Atlantic. NEA, Northeast Atlantic. WCA, Western Central Atlantic. SWA, Southwest Atlantic. WM, Western Mediterranean. NEP, Northeast Pacific. ECP, Eastern Central Pacific. SEP, Southeast Pacific. NWP, Northwest Pacific. WCP, Western Central Pacific. EIO, Eastern Indian Ocean.WIO, Western Indian Ocean. NA, not available.

a Reported as *Crossobothrium longicolle* (Molin, 1858).

b Reported as *Yamaguticestus* sp.

c Reported as *Yamaguticestus* sp.

d Reported as *Rhoptrobothrium myliobatidis* Shipley and Hornell, 1906.

e Reported as *Rhoptrobothrium* cf. *gambangi* Jensen & Caira, 2006.

f Reported as *Rhoptrobothrium gambangi* Jensen & Caira, 2006.

g Reported as *Myzocephaluss* sp.

h Reported as *Myzocephalus narinari* Shipley and Hornell, 1906.

i Reported as *Trilocularia gracilis*Olson et al., 2001.

j Host not listed in the source feature, typical definitive host *Squalus acanthias* Linnaeus.

### 2.4 Phylogenetic analysis

Phylogenetic analyses were performed using 11 newly generated sequences obtained from the plerocercoid larvae ex *T. sagittatus* and 47 homologous cestode sequences retrieved from GenBank, primarily representing adult specimens (Table 2). Alignments for each gene region were constructed separately in AliView (Larsson, 2014). The alignment was trimmed to the shortest sequence. Analysis of phylogeny based on Bayesian inference (BI) was performed using MrBayes 3.2.7 (Huelsenbeck and Ronquist, 2001; Ronquist et al., 2012). Test of best nucleotide substitution model was performed using PAUP (Swofford, 2002) implemented in MrModeltest 2.3 (Nylander, 2004). The BI was run with two random starting trees and four Markov chains, three heated and one “cold” for 2 x 10^∧^6 generations under the general time reversible model (GTR) with gamma distribution and invariable sites (G + I). Tree sampling was performed at 500 generations intervals. The first 500,000 generations were discarded as burn-ins. The Maximum Likelihood (ML) analyses was also used for comparison, was constructed in MEGA11, with 500 pseudoreplications. The best-fitting nucleotide substitution models for Maximum Likelihood (ML) analyses were selected in MEGA11 (Tamura et al., 2021). The best-fitting nucleotide substitution model for ML analysis was the General Time Reversible model with gamma-distributed rate variation among sites and a proportion of invariant sites (GTR + G + I).

## 3 Results

### 3.1 Occurrence and morphology of cestode larvae in *Todarodes sagittatus*

Cestode larvae were recovered from the digestive tract of 63 specimens of *T*. *sagittatus*, ranging in mantle length from 11 to 36 cm (mean 24 ± 5 cm). Of the examined squids, 31 were infected with cestodes, giving a prevalence of 46%. The intensity of infection ranged from 1 to 33 larvae per host, with a mean intensity of 7 ± 8. Female squids were significantly more infected than males (61% vs. 50%; χ^2^ = 6, df = 2, p = 0.05).

Inspection of the external surface of the digestive tract revealed no parasites. However, careful examination of the digestive lumen yielded a total of 220 plerocercoid larvae. Most larvae were located in the intestine (83%), where they moved freely, while a smaller proportion occurred in the stomach (14%). Only a few larvae were found in the caecal sac (2%) and caecum (1%).

**Fig. 1:**
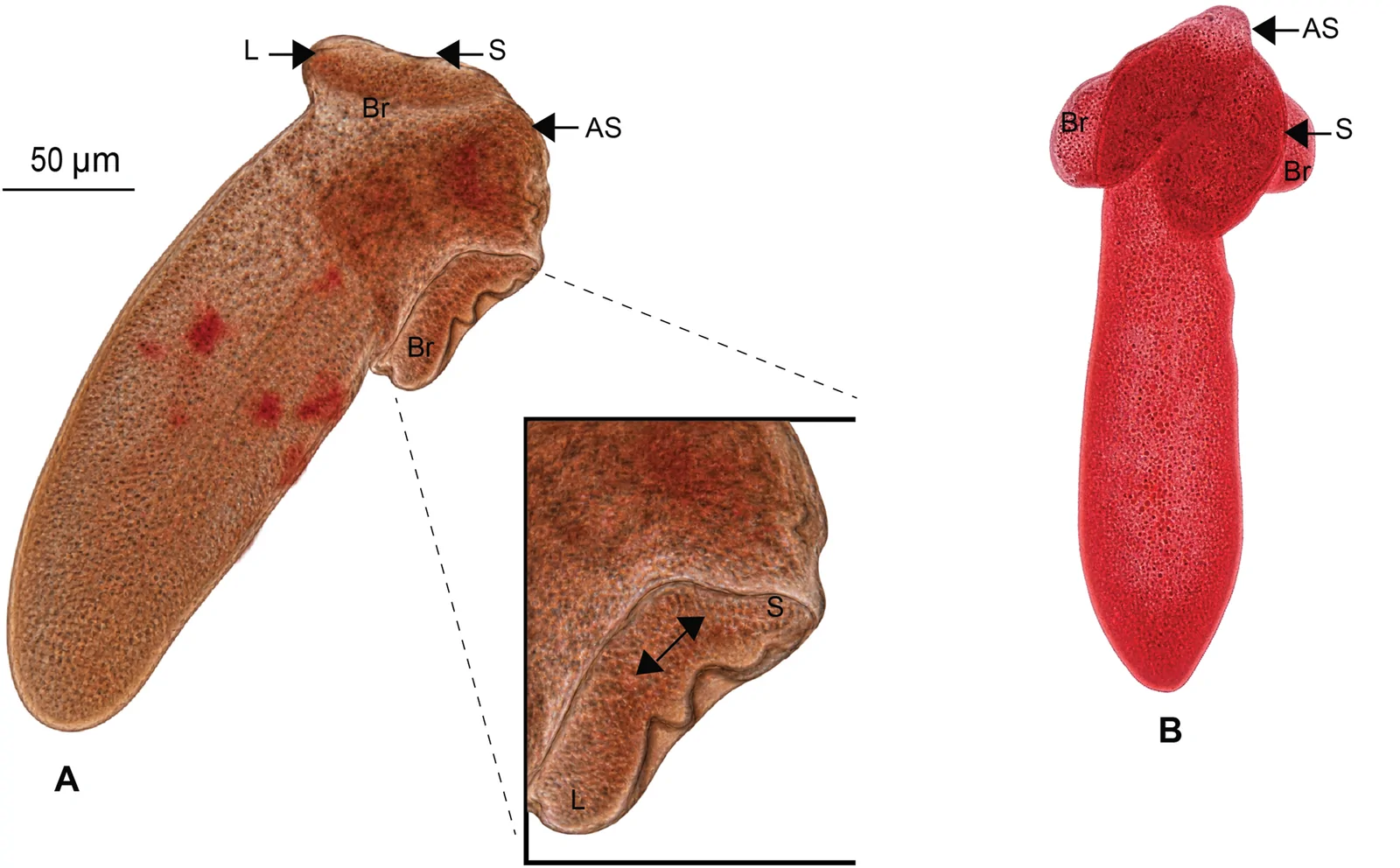
Phyllobothriidean cestode larvae recovered from the intestine of *Todarodes sagittatus*. **A.** Plerocercoid larva showing a scolex with an invaginated, cup-shaped accessory apical sucker (AS) and four bothridia (Br), each bearing an anterior sucker (S). The anterior sucker (S) is separated from the bothridial loculus (L) by a thin membrane (‣). **B**. Plerocercoid larva with the accessory apical sucker (AS) everted.

The larvae displayed morphological features consistent with members of the order Phyllobothriidea, as defined by Caira et al. (2014). All specimens showed a similar and relatively rudimentary morphology (Fig. 1), which prevented reliable identification to a lower taxonomic level. After removal from the digestive tract and transfer to Petri dishes, some larvae appeared contracted, with the scolex completely retracted, likely due to fixation or handling. The plerocercoids were elongated, oval in shape, and ended posteriorly in a rounded extremity. No specimens exhibited proglottids. The scolex was roughly triangular and bore four oval bothridia with free margins, each carrying a sucker on its anterior region (Fig. 1a). At the apex of the scolex, a cup-shaped apical accessory sucker was present (Fig. 1a). In some specimens, this apical sucker was everted and opened outward (Fig. 1b). The mean larval length was 1009 ± 57 μm (n = 6).

### 3.2 Phylogenetic analysis

Amplification of the D1–D3 region of the 28S rDNA from the 11 plerocercoid larvae yielded two distinct sequence types. The first sequence type, 1285 bp in length, was identical in seven larvae (Ts1.1–Ts1.7; GenBank accession numbers KU359304–KU359310). The second sequence type, 1281 bp long, was shared by the remaining four larvae (Ts2.1–Ts2.4; KU359311–KU359314). No sequence variation was detected within either group. The genetic divergence between the two larval lineages was low, corresponding to 1.7% across the aligned nucleotide sequence.

**Fig. 2:**
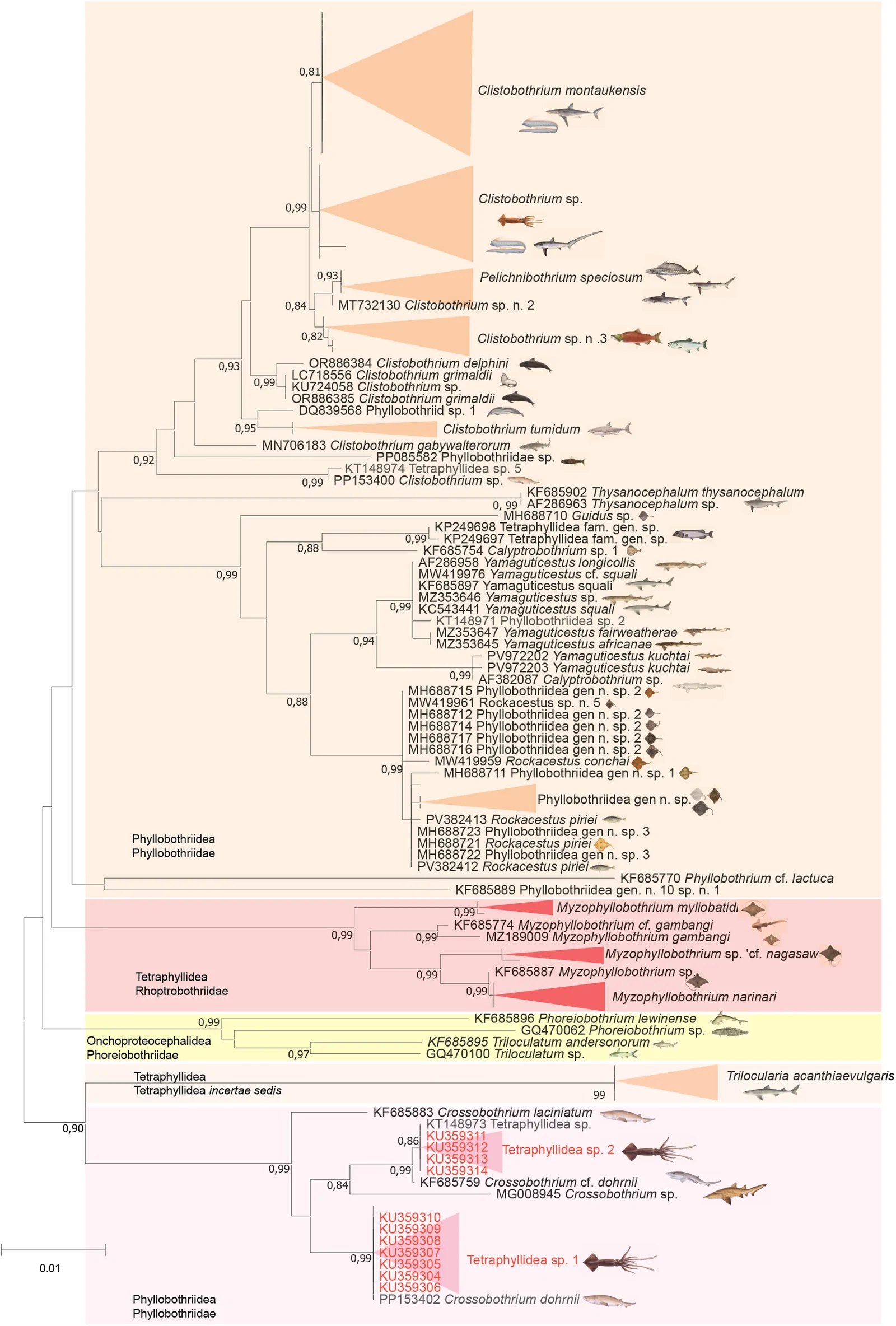
Bayesian inference phylogenetic tree based on partial 28S rDNA sequences showing the relationships between the 11 plerocercoid larvae recovered from *Todarodes sagittatus* (highlighted in red) and representative cestode taxa retrieved from GenBank. The larvae from the present study cluster within the *Crossobothrium* clade (Phyllobothriidea), forming two closely related groups corresponding to Tetraphyllidea sp. 1 and Tetraphyllidea sp. 2. Posterior probability values ≥ 0.70 are indicated at the nodes. Scale bar indicates the number of substitutions per site.

The phylogenetic analysis based on partial 28S rDNA sequences recovered several well-defined clades corresponding to previously recognized groups of elasmobranch cestodes (Fig. 2). The tree includes representatives of Phyllobothriidea, Rhoptrobothriidae, Onchoproteocephalidea, and taxa traditionally assigned to Tetraphyllidea *incertae sedis*.

Within Phyllobothriidea, several distinct clades are observed. A large assemblage includes species of *Clistobothrium* Dailey and Vogelbein, 1990, with sequences of *Clistobothrium montaukensis* Ruhnke, 1993 forming a well-supported group together with other *Clistobothrium* species. Other phyllobothriidean genera such as *Pelichnibothrium* Monticelli, 1888, *Yamaguticestus* Caira, Bueno & Jensen, 2021, *Rockacestus* Caira, Bueno & Jensen, 2021, and several unidentified phyllobothriidean taxa also appear as distinct lineages within this part of the tree. A separate lineage corresponds to members of the family Rhoptrobothriidae Caira, Jensen & Ruhnke, 2017, including species of *Myzophyllobothrium* Shipley and Hornell, 1906, which form a cluster clearly separated from the phyllobothriidean lineages. The tree also includes representatives of Onchoproteocephalidea, i.e. *Phoreiobothrium* Linton, 1889; *Triloculatum* Caira and Jensen, 2009, which form another distinct lineage. Near the base of the tree, taxa assigned to Tetraphyllidea *incertae sedis* are represented by *Trilocularia acanthiaevulgaris* Olson et al., 2001.

The sequences generated in the present study are located within the clade containing species of *Crossobothrium*. The first sequence type (Ts1) showed 100% identity with the adult cestode *Crossobothrium dohrnii* (Oerley, 1885) (PP153402), reported from the bluntnose sixgill shark *Hexanchus griseus* in the Gulf of Naples (Lopez-Verdejo et al., 2024). In the phylogenetic analysis, these sequences clustered with *C. dohrnii* within the *Crossobothrium* clade (Phyllobothriidea) (Fig. 2**)**. These larvae are therefore referred to hereafter as *C. dohrnii* (plerocercoid).

The second sequence type (Ts2) showed 100% identity with a plerocercoid larva previously reported from the southern shortfin squid *Illex coindetii* (Vérany) in Algerian waters (Tetraphyllidea gen. sp. 4NB-2015; KT148973) and 99% identity with the adult cestode *Crossobothrium* cf. *dohrnii* (KF685759) collected from the sharpnose sevengill shark *Heptranchias perlo* (Bonnaterre) in the Gulf of Mexico (Caira et al., 2014). In the phylogenetic tree, these sequences formed a closely related lineage within the *Crossobothrium* clade but did not correspond exactly to any described species. These larvae are therefore referred to hereafter as *Crossobothrium* sp. (plerocercoid).

## 4 Discussion

### 4.1 Taxonomic position of the larvae

The two larval lineages revealed by molecular analysis belong to the genus *Crossobothrium*. Since its establishment by Linton (1889), the taxonomy of this genus has undergone several revisions, and both its validity and species composition have long been debated (Southwell, 1925; Williams, 1968; Schmidt, 1986; Euzet, 1994). Early authors frequently confused *Crossobothrium* with morphologically similar genera such as *Phyllobothrium* Van Beneden, 1850, leading to uncertainty in species assignments and repeated changes in generic placement. A comprehensive revision of the genus by Ruhnke (2011) clarified its morphological diagnosis and restricted the number of valid species. At present, *Crossobothrium* comprises five recognized species parasitizing hexanchid sharks (Ruhnke et al., 2017).

One of the two larval groups recovered from *T. sagittatus* showed 100% sequence identity with the adult cestode *C. dohrnii* (PP153402), recently reported from the bluntnose sixgill shark *H. griseus* in the Tyrrhenian Sea off Naples (Lopez-Verdejo et al., 2024). This result strongly suggests that *T. sagittatus* acts as an intermediate or paratenic host for *C. dohrnii*. Historical records of this species are scarce. The only earlier mention dates back to Monticelli (1888), who reported a larva from *Ommastrephes todarus* (Lamarck) (a synonym of *T. sagittatus*) and attributed it to *Phyllobothrium dohrnii* (Oerley, 1885; Dollfus, 1923). However, that report relied on a limited description of the scolex and was based on a taxonomy that was still unsettled at the time. Confusion between species and even between the genera *Crossobothrium* and *Phyllobothrium* was common in early works (Southwell, 1925; Rees, 1946; Williams, 1968). Although Euzet (1959) recognized the validity of *Crossobothrium*, he later considered *C. laciniatum* Linton (1889) and *P. dohrnii* synonymous and did not retain *C. dohrnii* as a valid species in his later classification of Tetraphyllidea (Euzet, 1994).

More recent helminthological investigations of *T. sagittatus* have reported only unidentified cestode larvae referred to as *Scolex pleuronectis* Müller, 1788 (Nigmatullin et al., 2008). The present study therefore provides the first molecular identification of plerocercoid larvae infecting this host based on sequences of the D1–D3 region of the 28S rDNA.

### 4.2 Life-cycle implications and trophic transmission

Adult specimens of *C. dohrnii* have been reported exclusively from the spiral valve of hexanchid sharks, including *H*. *griseus* (Ruhnke, 1996; Lopez-Verdejo et al., 2024), the sharpnose sevengill shark *Heptranchias perlo* (Bonnaterre) (Palm and Schröder, 2001), and the broadnose sevengill shark *Notorynchus cepedianus* (Péron) (Alexander, 1963), which therefore represent its definitive hosts. Both *H. griseus* and *H. perlo* occur in Algerian waters (Hemida, 2005), although the latter is considered rare and is listed as threatened in the Mediterranean Red List (Cavanagh and Gibson, 2007; Ordines et al., 2011).

These sharks occupy the same ecological environment as *T. sagittatus*, a nectobenthic cephalopod that likely represents a potential prey item in coastal ecosystems. In the Mediterranean Sea, *T. sagittatus* has been reported among the stomach contents of both *H. perlo* (Capapé, 1980; Mulas et al., 2021) and *H. griseus* (Mulas et al., 2021). The occurrence of *C*. *dohrnii* plerocercoid larvae in the intestine of *T. sagittatus* therefore supports its role as an intermediate or paratenic host in the life cycle of this cestode. Overall, cephalopods are increasingly recognized as important trophic intermediates in the transmission of elasmobranch parasites. Their position as both active predators and frequent prey of sharks allows them to act as efficient vectors linking lower trophic levels with apex predators (Hochberg, 1990; Rohde, 2002; Coll et al., 2013). The presence of *Crossobothrium* plerocercoids in *T. sagittatus* therefore highlights the role of ommastrephid squids as key nodes in parasite transmission pathways within pelagic food webs.

Understanding the trophic web involving *T. sagittatus* may help clarify the life cycle of the cestodes it harbours. Like most cephalopods, *T. sagittatus* is an opportunistic predator that feeds on prey available in its immediate environment (Coll et al., 2013; Quetglas et al., 1999). Studies conducted in the northwestern Mediterranean, particularly around the Balearic Islands (an area adjacent to the Algerian Mediterranean Sea) show that its diet mainly includes the crustacean *Pasiphaea multidentate* Esmark, the amphipod *Phronima sedentaria* (Forskål), and myctophid fishes (Rosas-Luis et al., 2014). Crustaceans are known to act as first intermediate hosts harboring procercoid larvae (Busch et al., 2012).

**Fig. 3:**
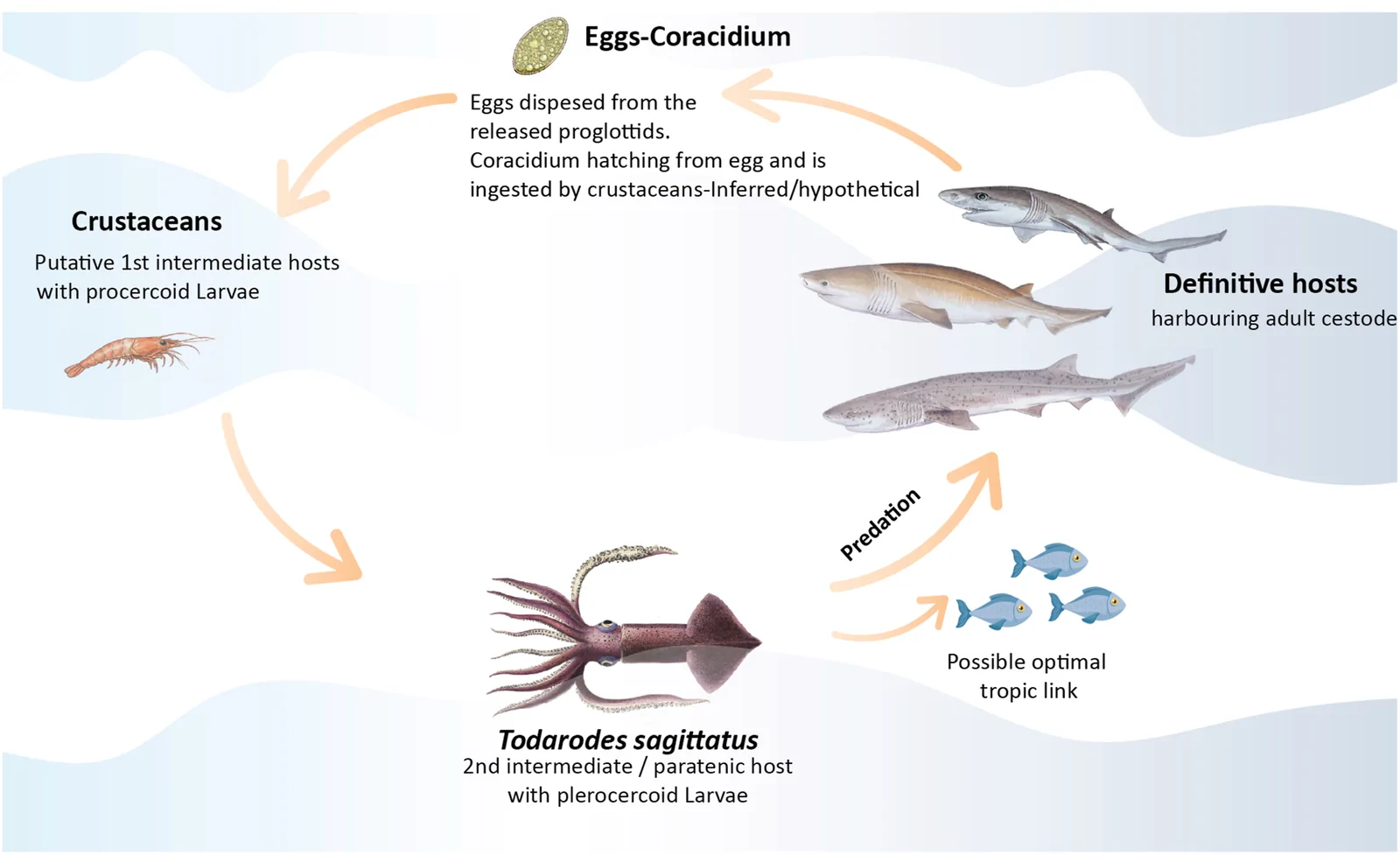
Proposed life cycle of *Crossobothrium dohrnii* (Oerley, 1885). Eggs released by hexanchid sharks (definitive hosts) hatch in the water and release coracidia. These infect a putative crustacean first intermediate host (e.g., *Pasiphaea multidentata*), in which procercoid larvae develop. The squid *Todarodes sagittatus* acquires infection through predation on infected crustaceans and harbours plerocercoid larvae as an intermediate or paratenic host. Hexanchid sharks become infected after feeding on infected squid. A fish host may occur as an optional trophic link between crustaceans and squid.

Because *T. sagittatus* hosts plerocercoid larvae, it likely acts as a second intermediate host or a paratenic host if a fish serves as the second intermediate host. The definitive host, hexanchid sharks such as *H. griseus* or *H. perlo* would acquire infection through predation on infected *T. sagittatus*. The plerocercoids would then develop into adult worms in the spiral valve, producing eggs that are released into the aquatic environment. Following egg formation and embryonation, a ciliated oncosphere (coracidium) forms within the eggshell; the coracidium is then released and swims in the water to attract the first intermediate host (planktonic crustaceans), thereby initiating a new cycle (Fig. 3).

### 4.3 Host sharing and parasite transmission among squids

The second larval lineage could not be assigned to a known (or recognized) species a specific species, although it clearly belongs to the genus *Crossobothrium*. Phylogenetic analysis showed that the sequences Ts2.1 (KU359311), Ts2.2 (KU359312), Ts2.3 (KU359313), and Ts2.4 (KU359314) cluster with the sequence *Tetraphyllidea* gen. sp. 4 NB-2015 (KT148973) in a single monophyletic group. This sequence corresponds to a plerocercoid larva previously collected from the squid *Illex coindetii* during an earlier helminthological study in Algerian waters (unpublished data). All five sequences are identical across the D1–D3 region of the 28S rDNA. This larval form therefore appears capable of infecting two different intermediate hosts, *I*. *coindetii* and *T*. *sagittatus*. These two ommastrephid squids occupy similar ecological niches, which likely facilitates overlap in their trophic networks (González et al., 2003). Given their comparable diets (Rosas-Luis et al., 2014), both species may act as alternative pathways for larval transmission, increasing the likelihood of successful infection of the definitive hosts *H. perlo* or *H. griseus*. Such host sharing among ecologically similar cephalopods may represent an important mechanism maintaining parasite circulation in pelagic ecosystems.

To our knowledge, this study represents only the second molecular characterization of cestode larvae from cephalopods in the Mediterranean Sea, the first having been conducted on the common octopus *Octopus vulgaris* Cuvier (Tedesco et al., 2020). Earlier work by Brickle et al. (2001) provided the first molecular characterization of plerocercoid larvae from a cephalopod (the patagonian squid *Doryteuthis* *gahi* (A. d'Orbigny) using the D2 region of 28S rDNA. More recently, Marmolejo-Guzmán et al. (2022) characterized cestode larvae belonging to the orders Trypanorhyncha and Onchoproteocephalidea from the Mexican four-eyed octopus *Octopus maya* Voss & Solís in Yucatán, Mexico. Guardone et al. (2020) also applied molecular tools to characterize plerocercoids from the longfin inshore squid *Doryteuthis pealeii* (Lesueur) in Atlantic waters. Similar records from the Pacific Ocean include plerocercoid larvae of the tentaculariid Nybelinia enterika Zoral & Lajbner *in* Zoral et al. (2025), and the phoreiobothriid *Phoreiobothrium* sp. from the oval squid *Sepioteuthis lessoniana* Lesson. in coastal waters of Okinawa Island (northwest Pacific). The tentaculariid *N. surmenicola* Okada *in* Dollfus, 1929) has also been reported from the Japanese flying squid *T*. *pacificus* (Steenstrup) in the East Sea (Korea, northwest Pacific) (Lee et al., 2016), further highlighting the diversity of cestode larvae infecting cephalopods.

Despite the ecological importance of cephalopods in marine food webs, molecular identification of their parasites remains relatively scarce. Many earlier records relied exclusively on morphological identification of larval forms, which are often taxonomically ambiguous. Integrative approaches combining morphology, molecular data, and phylogenetic analyses are therefore essential for clarifying parasite diversity and life cycles (Blasco-Costa and Poulin, 2017).

## 5 Conclusion

This study contributes to the understanding of cestode diversity and life cycles in Mediterranean cephalopods, particularly in *Todarodes sagittatus*. By combining morphological observations with molecular characterization of the D1–D3 domains of the 28S rDNA, we were able to identify the plerocercoid stage of *Crossobothrium dohrnii* in this squid species, confirming its role as an intermediate or paratenic host in the life cycle of this elasmobranch parasite. In addition, a second larval lineage was detected which, although not assignable to a described species, showed clear phylogenetic affinity with a larva previously reported from *Illex coindetii*. This finding suggests that multiple ommastrephid squids may participate in the transmission of closely related *Crossobothrium* species, reflecting a more complex trophic network than previously recognized. Additionally, our results highlight the ecological importance of cephalopods as intermediate links in the transmission of cestodes to their definitive hosts, hexanchid sharks. Future studies incorporating additional molecular markers, broader host sampling, and detailed ecological data will likely reveal a greater diversity of cestodes infecting Mediterranean cephalopods and further improve our understanding of parasite circulation within marine ecosystems.

## CRediT authorship contribution statement

**Nourreddine Benyahia:** Writing – original draft, Methodology, Investigation, Funding acquisition, Formal analysis, Data curation, Conceptualization. **Chahinez Bouguerche:** Writing – review & editing, Writing – original draft, Formal analysis, Data curation. **Nadia Kechemir-Issad:** Writing – review & editing, Writing – original draft, Supervision, Resources, Project administration, Methodology, Investigation, Funding acquisition, Formal analysis, Data curation, Conceptualization.

## Declaration of competing interest

None.

## Footnotes

1 Specimens were fixed in Bouin's solution at the time of collection in 2005, following the protocol then in use. Note that fixative is not recommended for morphological studies of cestode larvae; see Jensen and Bullard (2010) for standard processing methods.
